# Population exposure–response analysis of cabozantinib efficacy and safety endpoints in patients with renal cell carcinoma

**DOI:** 10.1007/s00280-018-3579-7

**Published:** 2018-04-17

**Authors:** Steven Lacy, Jace Nielsen, Bei Yang, Dale Miles, Linh Nguyen, Matt Hutmacher

**Affiliations:** 1 Exelixis Inc., 210 East Grand Avenue, South San Francisco, CA 94080-0511 USA; 2 Ann Arbor Pharmacometrics Group, Inc., Ann Arbor, MI USA

**Keywords:** Cabozantinib, Exposure–response modeling, Renal cell carcinoma

## Abstract

**Background:**

In the phase III METEOR trial, tyrosine kinase inhibitor cabozantinib significantly improved progression-free survival (PFS), objective response rate (ORR), and overall survival compared to everolimus in patients with advanced renal cell carcinoma (RCC) who had received prior VEGFR inhibitor therapy. In METEOR, RCC patients started at a daily 60-mg cabozantinib tablet (Cabometyx™) dose but could reduce to 40- or 20-mg to achieve a tolerated exposure.

**Objectives and methods:**

Exposure–response (ER) models were developed to characterize the relationship between cabozantinib at clinically relevant exposures in RCC patients enrolled in METEOR and efficacy (PFS and tumor response) and safety endpoints.

**Results:**

Compared to the average steady-state cabozantinib concentration for a 60-mg dose, exposures at simulated 40- and 20-mg starting doses were predicted to result in higher risk of disease progression or death [hazard ratios (HRs) of 1.10 and 1.39, respectively], lower maximal median reduction in tumor size (− 11.9 vs − 9.1 and − 4.5%, respectively), and lower ORR (19.1 vs 15.6 and 8.7%, respectively). The 60-mg exposure was also associated with higher risk for selected adverse events (AEs) palmar-plantar erythrodysesthesia syndrome (grade ≥ 1), fatigue/asthenia (grade ≥ 3), diarrhea (grade ≥ 3), and hypertension (predicted HRs of 2.21, 2.01, 1.78, and 1.85, respectively) relative to the predicted average steady-state cabozantinib concentration for a 20-mg starting dose.

**Conclusion:**

ER modeling predicted that cabozantinib exposures in RCC patients at the 60-mg starting dose would provide greater anti-tumor activity relative to exposures at simulated 40- and 20-mg starting doses that were associated with decreased rates of clinically relevant AEs.

**Electronic supplementary material:**

The online version of this article (10.1007/s00280-018-3579-7) contains supplementary material, which is available to authorized users.

## Introduction

Renal cell carcinoma (RCC) accounts for approximately 2–3% of all malignancies in adults with about one-third of patients having metastatic disease at diagnosis [1–3]. Recent advances in the treatment of RCC have been made based on improved understanding of the molecular biology of this disease, including the involvement of pathways linked to the vascular endothelial growth factor receptor (VEGFR), mammalian target of rapamycin (mTOR), and the programmed cell death (PD-1) receptor [4–6]. Therapeutic approaches for treatment of RCC include the VEGF antibody bevacizumab, mTOR inhibitors temsirolimus and everolimus, the PD-1 checkpoint inhibitor nivolumab, and tyrosine kinase inhibitors (TKI) such as sunitinib, pazopanib, sorafenib, levatinib, and axitinib [7]. Although these therapies were significant advancements in the treatment of RCC, disease progression is common as resistance to these treatments eventually develops.

Cabozantinib is an inhibitor of receptor tyrosine kinases including VEGFR2, and the tyrosine kinases MET (hepatocyte growth factor receptor) and AXL (GAS6 receptor) implicated in development of resistance to RCC therapy [8–10]. In the pivotal phase III METEOR study, cabozantinib improved overall survival, decreased disease progression, and increased objective response in patients with advanced RCC who had received prior VEGFR-TKI treatment [11]. The cabozantinib tablet formulation (Cabometyx™) is approved at a 60-mg-free-base equivalent (FBE) daily dosage for the treatment of patients with advanced RCC in USA who have received prior anti-angiogenic therapy and in the European Union (EU) following prior VEGF-targeted therapy, with dosage adjustments to 40-mg FBE and then 20-mg FBE permitted to manage adverse events (AEs) [12, 13]. In METEOR, 60% of patients treated with cabozantinib had at least one dose reduction and 70% required dose modification (i.e., dose interruption, reduction, or increase). The most frequent AEs leading to dose reduction were diarrhea (16%), palmar-plantar erythrodysesthesia syndrome (PPES; 11%), fatigue (10%), and hypertension (7.6%).

Cabozantinib capsule formulation (Cometriq^®^) is approved at a dose of 140-mg FBE in USA for treatment of patients with progressive, metastatic medullary thyroid cancer (MTC), and in the EU for the treatment of progressive, unresectable locally advanced or metastatic MTC, with dose reductions to 100-mg FBE then to 60-mg FBE to manage AEs [14, 15]. Exposure–response (ER) modeling in MTC patients showed an increased risk of time to the first dose modification to be highly correlated with lower cabozantinib apparent clearance (CL/*F*) values; however, no clear association was observed between time to first dose modification and progression-free survival (PFS) [16]. In an integrated population PK (popPK) analysis, MTC cancer type was shown to be a statistically significant covariate on cabozantinib CL/*F*, with values approximately twofold higher relative to patients with other types of malignancies (including RCC) [17]. Several possible factors may underlie the higher cabozantinib clearance observed in MTC patients, including differences in the incidence or severity of treatment-related diarrhea or hypocalcemia, or use of concomitant medications; however, an exact cause has yet to be identified [17]. The current ER analyses were thus undertaken to better understand the relationship between cabozantinib exposure and efficacy, safety, and the need for dose adjustments in RCC patients.

## Methods

### Study design and data

The ER analyses were conducted utilizing data from the phase III METEOR study of cabozantinib in patients with RCC [11]. METEOR was a multicenter, randomized, controlled trial of cabozantinib [60-mg FBE once a day (QD)] versus everolimus (10-mg QD), with PFS as the primary endpoint, and overall survival (OS) and objective response rate (ORR) as secondary endpoints. A total of 658 patients were randomized 1:1 to receive cabozantinib (*n* = 330) or everolimus (*n* = 328). Randomization was stratified by number of prior VEGFR-TKI therapies and number of risk factors per Memorial Sloan-Kettering Cancer Center (MSKCC) criteria. Patients were ≥ 18 years of age with advanced or metastatic RCC with a clear-cell histology and measurable disease per RECIST. To manage AEs, dose modifications were allowed that included dose interruptions and reductions. The dose of cabozantinib could be reduced to 40-mg and then to 20-mg from the starting dose of 60-mg. The dose of everolimus could be reduced to 5-mg and then to 2.5-mg. Radiographic assessments were performed at screening and every 8 weeks for the first year, and every 12 weeks thereafter. Safety was assessed in all patients who received at least one dose of study drug every 2 weeks for the first 8 weeks and every 4 weeks thereafter. At the discretion of the Investigator, treatment could be continued after radiographic progression. One blood sample for plasma cabozantinib concentration determinations was collected at approximately 8 or more hours after the prior evening’s dose on day 29 and day 57 of the study.

### Bioanalytical methods

Plasma cabozantinib concentrations were measured using a validated liquid chromatographic–tandem mass spectrometry method. The lower limit of quantitation was 0.5 ng/mL [18].

### Software and modeling strategy

Time-to-event Cox proportional hazard (PH) models were developed using SAS Version 9.3 (SAS Institute Inc., Cary NC). Longitudinal tumor size and time-to-event dose modification models were developed using NONMEM version 7.3 (ICON Development Solutions, Ellicott City, MD). The stochastic approximation expectation maximization (SAEM)/importance sampling (IMP) estimation method was used for the tumor size model and the Laplacian estimation method was used for the repeated time-to-event dose modification model. Differences in objective function values (OFVs) between competing models as well as standard goodness-of-fit plots were used to identify the best fitting model among those evaluated.

### Population pharmacokinetic model

A popPK model was developed from nine clinical studies, comprised of 1534 subjects. The detailed method for the popPK model development is described in a separate manuscript [17]. Individual exposures for subjects in the METEOR study were predicted from this popPK model for use as the exposure metrics for the present ER analysis.

### Exposure response analysis for time-to-event endpoints

#### Progression-free survival and safety endpoints

Time-to-event analyses were performed to characterize the ER relationship between cabozantinib exposure and clinical endpoints including PFS and six safety endpoints. The number of patients with events and the total number of patients at risk are listed in Supplemental Table 1. The safety endpoints evaluated included fatigue/asthenia (grade ≥ 3), PPES (grade ≥ 1), nausea or vomiting, diarrhea (grade ≥ 3), hypertension (systolic BP > 160 mmHg or diastolic BP > 100 mmHg), and stomatitis (grade ≥ 3).

The extended Cox PH model was used to describe the relative hazard for all endpoints (efficacy, safety, and dose modification) and to allow for time-varying cabozantinib exposure. The Cox PH model is represented by the equation:

1 $$h(t,X(t))={h_o}(t) \cdot \exp (\beta \cdot X(t)),$$

where *h*(*t, X*(*t*)) denotes the hazard at time *t, h*_*o*_(*t*) is the background hazard function, *β* is a vector of the regression coefficients, and *X*(*t*) is a matrix of covariates which may vary with time. A Cox PH model was fit for each endpoint and three possible cabozantinib exposure measures: average concentration (*C*_avg_) over a pre-specified time interval ranging from 1 day to 3 weeks prior to time *t* or from time 0 to time *t* or area-under-the-plasma-concentration–time-curve from time 0 to time *t* (AUC0_0–*t*_).

The impact of cabozantinib exposure on the relative hazard was evaluated during base model development. Both linear (Eq. 2) and nonlinear (Eq. 3) functional forms were evaluated:

2 $$h(t,{X_{{\text{ex}}}}(t))={h_o}(t) \cdot \exp ({\beta _{{\text{ex}}1}} \cdot {X_{{\text{ex}}}}(t)),$$

3 $$h(t,X_{{{\text{ex}}}}^{\prime }(t))={h_o}\left( t \right) \cdot \exp ({\beta _{{\text{ex2}}}} \cdot X_{{{\text{ex}}}}^{\prime }(t));~X_{{{\text{ex}}}}^{\prime }(t)=\frac{{{X_{{\text{ex}}}}(t)}}{{{X_{{\text{ex}}}}(t)+{\text{E}}{{\text{C}}_{50}}}},$$

where *X*_ex_(*t*) or *X*′_ex_(*t*) is time-varying cabozantinib exposure measure, *β*_ex1_ represents the slope in the log-linear model, *β*_ex2_ represents the maximum drug effect in the *E*_max_ model, and EC_50_ represents a range of fixed values for the exposure at which half of the maximal effect is achieved.

Covariate effects were evaluated only for PFS using the SELECTION = SCORE option in the PHREG procedure within SAS. Schwarz’s Bayesian Criterion (SBC) was calculated to find the most parsimonious or final model. A summary of the covariates evaluated is provided in Supplemental Table 2. The final model was used to evaluate the importance of selected cabozantinib exposures on the rate of events in the survival/probability scale.

#### Longitudinal sum of tumor diameter model

Nonlinear mixed-effects modeling was used to develop a model to describe the relationship between longitudinal sum of tumor diameter measurements and *C*_avg_. The sum of tumor diameter over time is described in Eq. 4 [19]:

4 $${\text{d}}Y/{\text{d}}t={\text{Growth}} - {\text{Drug}}\;{\text{effect,}}$$

where ${\text{d}}Y/{\text{d}}t$ is the change in tumor diameter over time, and growth represents the increase of tumor diameter over time, which is independent of any drug effects. Drug effect represents the first-order drug induced decay. Linear and nonlinear relationships were evaluated between cabozantinib and decay rate. In addition, models accounting for resistance were also tested.

The model with the best fit to the data had a first-order growth rate, nonlinear cabozantinib drug effect, and a resistance component. The model included exponential error models for inter-individual variability (IIV) and an additive error model for residual variability. The model is defined in Eq. 5:

5 $$\frac{{{\text{d}}Y}}{{{\text{d}}t}}={k_{{\text{grow}}}} \cdot Y - ~\frac{{(({k_{{\text{dmax~}}}}+~{k_{{\text{dmaxtot~}}}} \cdot {{\text{e}}^{ - {k_{{\text{tol}}}} \cdot t~}})~ \cdot {C_{{\text{avg}}}}}}{{({\text{E}}{{\text{C}}_{50}}~+{C_{{\text{avg}}}})}}~ \cdot ~Y,$$

where d*Y*/d*t* is the change in tumor diameter per unit time, *k*_grow_ is the first-order growth rate constant, *k*_dmax_ is the maximum non-attenuating drug induced tumor decay rate, *k*_dmaxtot_ governs the maximum loss in the decay rate due to resistance, *k*_tol_ is the rate constant which governs the rate of attenuation, EC_50_ is the cabozantinib concentration yielding one-half of the current tumor decay rate, and *C*_avg_ is the individual predicted daily average cabozantinib concentration.

#### Dose modification

The relationship (relative risk) between individual predicted cabozantinib CL/*F* and the rate of dose modifications (i.e., reductions or interruptions) was assessed using the linear model (Eq. 2) and the nonlinear model(s) (Eq. 3). As CL/F was log normally distributed, a linear model using log-transformed CL/*F* was also evaluated.

Repeated time-to-event analyses were also performed to characterize the ER relationship between cabozantinib *C*_avg_ and the dose modification of any kind (DMAK) that considered dose escalations, reductions, and interruptions necessary for realistic simulations (Eq. 6).

The date of the first dose of study medication represented “time 0” in these analyses. *C*_avg_ was used in these models. The number of events in a particular patient ranged from 0 to 52. The hazard for the DMAK model is defined by the following equation:

6 $$\lambda =\exp ({\theta _{{\text{base}}}}+~{\theta _{{\text{drug}}}} \cdot {C_{{\text{avg}}}})\quad \{ {\text{if}}\;{\text{dose}}>0\} ,$$

$$\lambda =\exp ({\theta _{{\text{base}}}} - {\theta _{{\text{base-hold}}}})\quad \{ {\text{if}}\;{\text{dose}}=0\} ,$$

where ${\theta _{{\text{base}}}}$ represents the baseline log hazard, ${\theta _{{\text{drug}}}},$ the change in log hazard per unit cabozantininb concentration, and ${\theta _{{\text{base-hold}}}},$ the baseline log hazard for dose hold. The hazard was dependent on whether a patient was currently on a dose interruption (i.e., dose = 0). Cabozantinib exposure was tested on the hazard for dose interruption, but this did not result in a reduction in the OFV and was not included in the final model. The baseline hazard during dose interruptions is larger than the hazard during active treatment indicating that there is an increased risk of a dose modification during dose interruptions. This is consistent with the relatively short duration of the majority of dose interruptions. When subjects are not on a dose interruption, increases in cabozantinib concentration increase the instantaneous risk for a dose modification. The parameter estimates for the DMAK model are shown in Supplemental Table 3.

### Simulations

Simulations were performed to compare the effects of cabozantinib on longitudinal tumor size for a starting dose of 60-, 40-, and 20-mg. First, predictions for DMAK analysis were based on Monte Carlo simulations (1000 patients) for trial duration of 365 days. Uncertainty in the predictions was not computed. *C*_avg_ was calculated interactively based on dosing change predicted from the DMAK model. The tumor model was used to predict the change in tumor size over time for the 1000 subjects at 60-, 40-, and 20-mg starting dose groups. The median tumor diameter was tabulated by day for each treatment group.

## Results

### Population pharmacokinetic model

A two-compartment model with the first-order elimination and a dual absorption (first order + zero order) process adequately described the observed cabozantinib concentration data. The results of the model and the covariates analyses are described in a separate manuscript [17]. The predicted exposure measures used in the ER analyses in this report were derived from the post hoc PK parameters from this popPK model.

### Cox proportional hazard models for progression-free survival

**Fig. 1:**
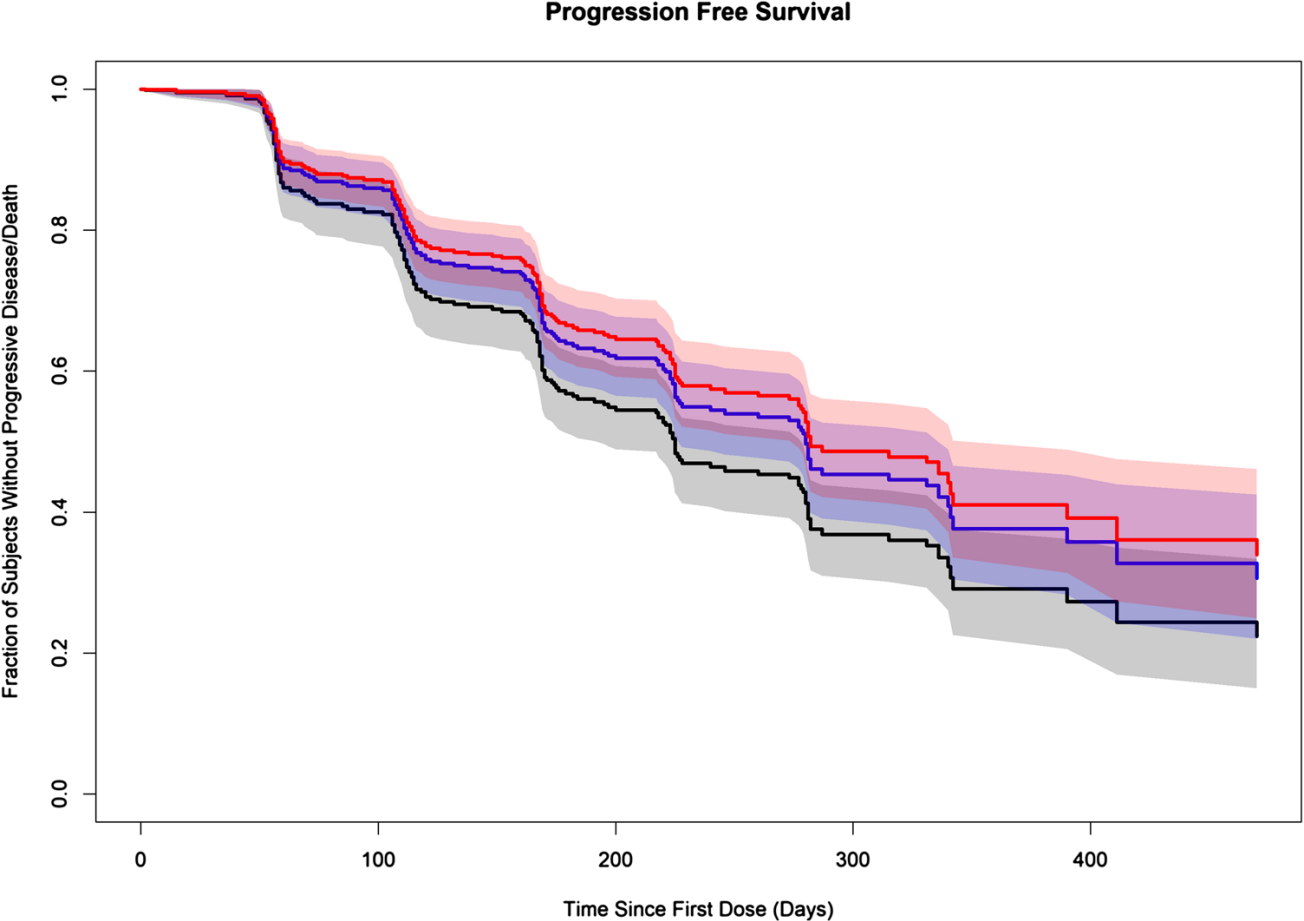
Predicted survival curves for progression-free survival for selected values of average cabozantinib concentration. Typical individual predicted steady-state average cabozantinib concentration for the 20-mg (black), 40-mg (blue), and 60-mg (red) doses are 375, 750, and 1125 ng/mL, respectively. The solid line represents the fraction of subjects at each dose level without progress disease or death over time. The shaded areas represent 95% confidence intervals

A total of 315 patients were included in PFS analysis. Different cabozantinib exposure measures were fit initially to a linear Cox PH model; the time-varying average cabozantinib concentration calculated over the 3 weeks prior to the event *t* (*C*_avg3w_) resulted in the lowest partial (negative 2 log) likelihood (− 2LL) value and was the only statistically significant exposure measure (*p* < 0.001). The relationship between cabozantinib *C*_avg3w_ and PFS was further assessed using nonlinear models over a range of EC_50_ values for *C*_avg3w_ (50–300 ng/mL). The nonlinear models resulted in statistically significant reductions in − 2LL compared to the linear model; an EC_50_ value of 100 ng/mL resulted in the best model fit and was used to illustrate the relationship between disease progression and cabozantinib concentrations. Figure 1 illustrates the impact of selected cabozantinib exposure values on the predicted survival curves for PFS. These curves show the predicted fraction of subjects without disease progression or death. The typical individual predicted average steady-state cabozantinib concentration for 20-mg (375 ng/mL), 40-mg (750 ng/mL), and 60-mg (1125 ng/mL) QD doses was used for exposure in the predictions and the predictions did not account for dose holds. Relative to reference steady-state plasma concentration at 60-mg, cabozantinib concentrations at lower simulated starting doses of 40- and 20-mg yielded hazard ratios (HRs) (95% lower–upper confidence limits) for disease progression or death [1.10 (1.07–1.12) and 1.39 (1.29–1.49), respectively] indicating an increased risk with decreasing plasma drug concentrations.

**Fig. 2:**
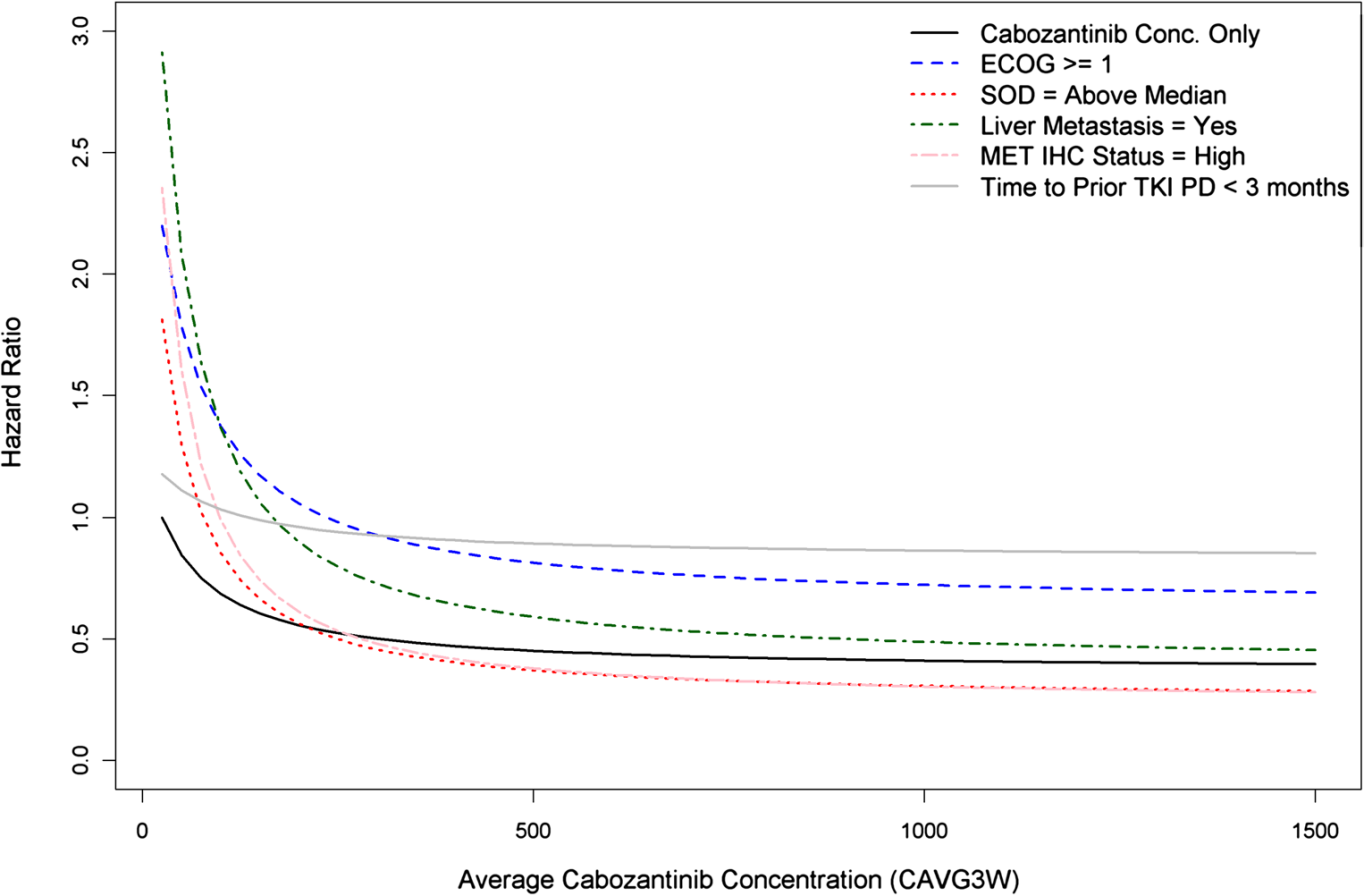
Comparison of predicted hazard ratio for different covariate effects for final progression-free survival model. The reference hazard represents an average cabozantinib concenration equal to 25 ng/mL (EC_20_), an ECOG score equal to zero, a baseline sum of tumor diameter that is below the median, no liver metastasis, an MET IHC status equal to low, and a time to progressive disease greater than or equal to 3 months for the previous TKI therapy. *EC*_*20*_ drug exposure producing 20% of the maximum effect, *ECOG* Eastern Cooperative Oncology Group, *MET IHC* hepatocyte growth factor receptor protein immunohistochemistry, *SOD* sum of diameters, *TKI* tyrosine kinase inhibitor, *PD* progressive disease

The most parsimonious model which was selected as the final model contained covariates of cabozantinib *C*_avg3w_, baseline Eastern Cooperative Oncology Group (ECOG) score ≥ 1, baseline sum of tumor diameter greater than the median, liver metastasis, high MET immunohistochemistry (IHC) status, and elapsed time less than 3 months before progressive disease on prior TKI therapy. The parameter estimates for the final PFS model are listed in Supplemental Table 4. The impact of each covariate is pictured in Fig. 2. Patients with increasing cabozantinib concentrations were predicted to have a decreased risk of progressive disease or death. Patients with baseline ECOG score ≥ 1, baseline sum of tumor diameter above median, liver metastasis, and high MET IHC status were predicted to have a higher baseline hazard ratio of progressive disease or death, but these effects were largely offset by higher cabozantinib exposure. At higher cabozantinib concentrations, the HR for PFS was similar with or without these covariates.

### Longitudinal tumor growth

A total of 319 patients with 1637 evaluable tumor diameter measurements were included in the analysis. The parameter estimates for the final tumor growth model is in Supplemental Table 3. The attenuation half-life was 25.6 days for a typical patient, indicating that this component of tumor decay rate was near zero by 128 days. The EC_50_ is 251 ng/mL and the EC_80_ value is about 1000 ng/mL, suggesting that the recommended 60-mg daily dosage was near the plateau of the dose–response curve as cabozantinib *C*_avg_ for a 60-mg QD dose is 1125 ng/mL. Baseline tumor size variability between patients was large (CV = 72%).

### Cox proportional hazard models for safety endpoints

**Fig. 3:**
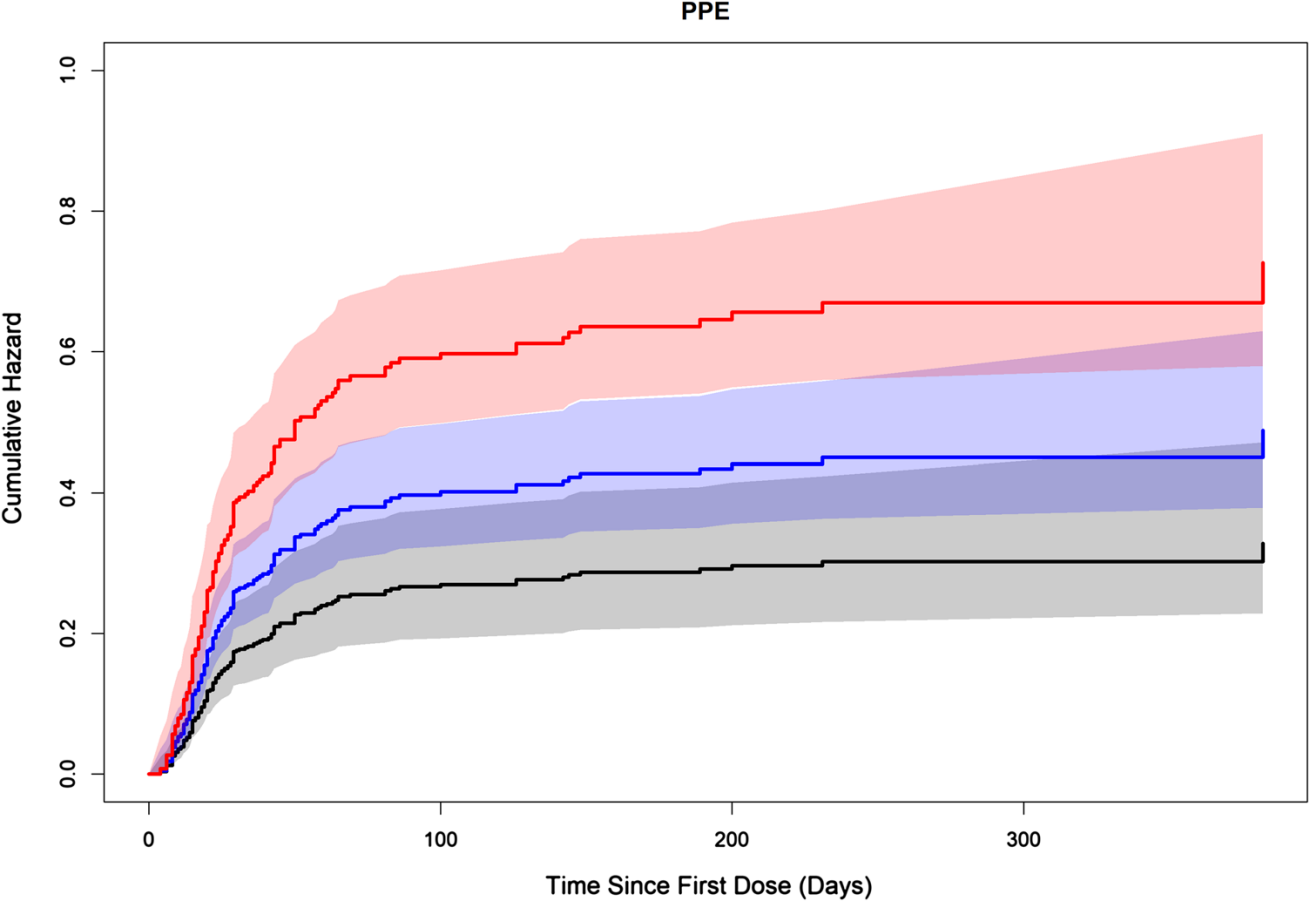
Predicted accumulative hazards for palmar-plantar erythrodysesthesia (PPE) syndrome at specific, constant average cabozantinib concentrations. Typical individual predicted steady-state average cabozantinib concentration for the 20-mg (black), 40-mg (blue), and 60-mg (red) doses is 375, 750, and 1125 ng/mL, respectively. The solid line represents the accumulative hazard for PPE at each dose level over time. The shaded areas represent 95% confidence intervals

For safety endpoints fatigue/asthenia, PPES, nausea/vomiting, and diarrhea, the time-varying average cabozantinib concentration over 2 weeks prior to time *t* (*C*_avg2w_) resulted in the lowest − 2LL. For hypertension and stomatitis, the time-varying average concentration over the 24 h prior to time *t* (*C*_avg1d_) and concentration calculated from time zero to time equal to *t* (*C*_avg0t_) resulted in the lowest − 2LL, respectively. Parameter estimates for the final models for each of these AEs are shown in Supplemental Table 4. An increase in average cabozantinib concentrations was associated with increased risk of PPES (grade ≥ 1; Fig. 3), fatigue/asthenia (grade ≥ 3), hypertension (systolic blood pressure > 160 mmHg or diastolic blood pressure > 100 mmHg), and diarrhea (grade ≥ 3). The predicted HRs (95% lower–upper confidence limits) for PPES, fatigue/asthenia, hypertension, and diarrhea were 2.21 (1.60, 3.06), 2.01 (1.22, 3.31), 1.85 (1.33, 2.57), and 1.78 (1.08, 2.91), respectively, based on the predicted steady-stage average cabozantinib concentration for a 60-mg dose relative to a 20-mg dose. For these respective AEs, lower predicted HRs (95% lower–upper confidence limits) were also evident based on the predicted steady-stage average cabozantinib concentration for a 60-mg dose relative to a 40-mg dose [i.e., 1.49 (1.27, 1.75), 1.42 (1.11, 1.82), 1.36 (1.15, 1.60), and 1.33 (1.04, 1.70)]. Statistically significant ER relationships were not found for nausea/vomiting (grade ≥ 3) or stomatitis (grade ≥ 3), but the frequencies of events for these two endpoints were small (*n* = 16 for nausea/vomiting and *n* = 10 for stomatitis).

### Dose modifications

317 patients were included in the analysis of dose modifications. The − 2LL for the linear model using log-transformed CL/*F* (log-linear CL/*F* model) was lower than the linear CL/*F* model and was similar to the − 2LL for the best nonlinear model. Residual diagnostics were similar for the linear model using log CL/*F* and the best nonlinear model. Overall, the log-linear CL/*F* was selected over the best nonlinear model.

**Fig. 4:**
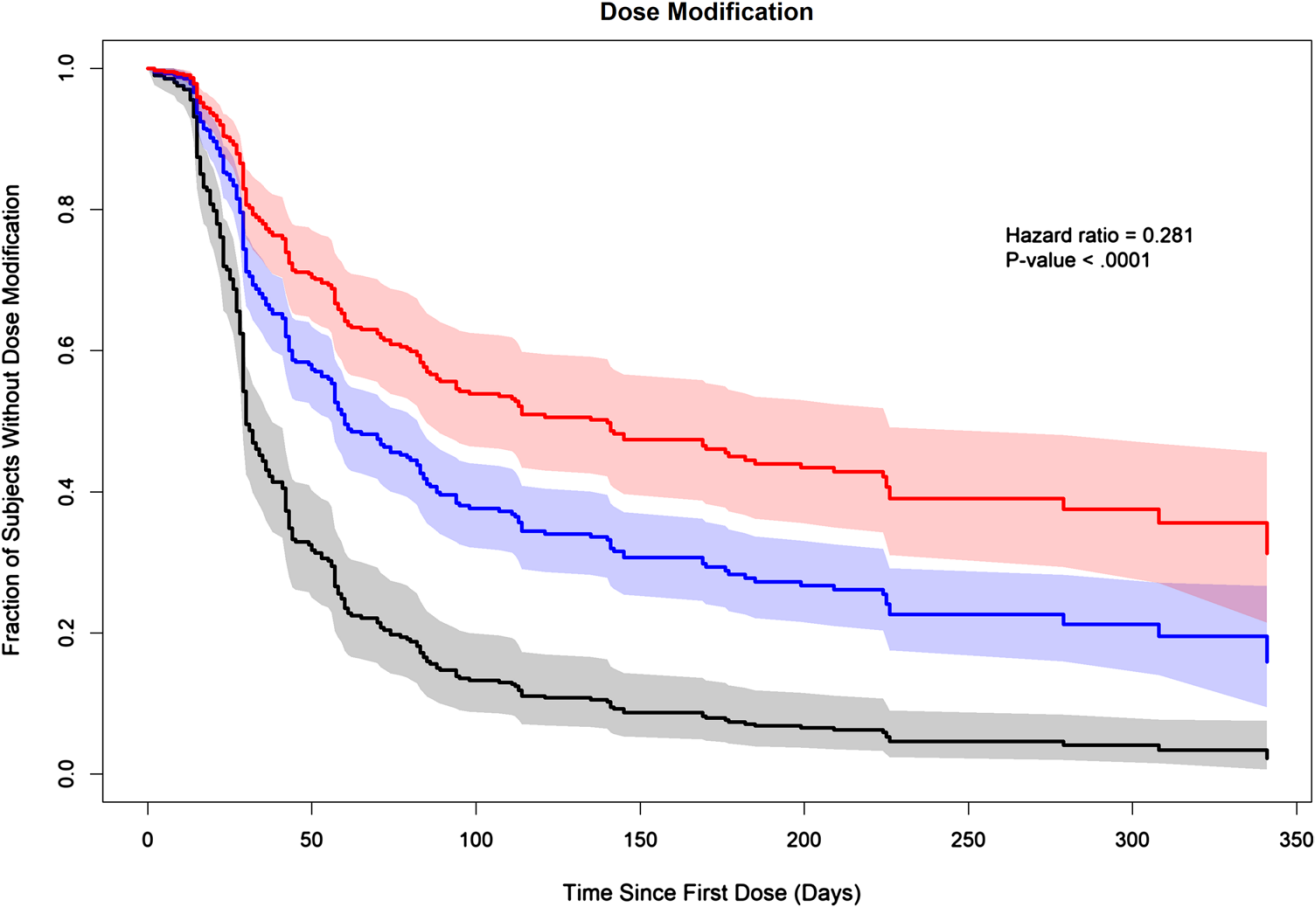
Predicted fractions of subjects without dose modification for selected values of cabozantinib apparent clearance. The solid black line (shaded black areas represent 95% CI) represents the fraction of subjects without dose modification over time for CL/*F* of 1.3 L/h, the solid blue line (shaded blue areas represent 95% CI) represents the fraction of subjects without dose modification over time for CL/*F* of 2.3 L/h, and the solid red line (shaded red areas represent 95% CI) represents the fraction of subjects without dose modification over time for CL/*F* of 3.3 L/h. The hazard ratio of 0.281 (*p* < 0.0001) indicates 0.281 times less risk of dose modification for one unit increase of ln(CL)

A statistically significant relationship was identified between individual predicted cabozantinib CL/*F* and the relative risk of dose modifications (*p* value < 0.0001). The parameter estimate for the dose modification model [*β*_CL/*F*_ (SE) = − 1.27033 (0.177930)], indicating that the log HR decreases with increasing log CL/F, was used to compute the relative HR for different values of CL/F. Relative to a CL/F of 2.3 L/h, the HR (95% CI) for risk of dose modification for a lower CL/*F* value of 1.3 L/h was approximately two times greater [2.07 (1.60, 2.52)]. Figure 4 illustrates the impact of selected cabozantinib CL/*F* values on the predicted survival curves; these curves show the predicted fraction without dose modifications over time for CL/*F* values of 1.3, 2.3, and 3.3 L/h.

### Simulations

First, the dose modification model DMAK was used to simulate longitudinal *C*_avg_ for 1000 patients over a 12-month period based on the changing events. To mimic dose change scenarios in the observed data set, at the time of each simulated event, the observed probability of a dose reduction, interruption, or escalation based on the current dose was used. If the current dose was 0 (representing a dose interruption), the observed probability of escalating to 20-, 40-, or 60-mg given the dose prior to the interruption was used. The percentage of patients that were predicted to be on the 20-mg, 40-mg, and 60-mg dosages after 6 and 12 months for the observed data set for 60-mg and for the simulated 60-mg and simulated 40-mg starting dose data sets are shown in Supplemental Table 5.

The simulated 60-mg starting dose yielded similar percentages of patients that dose reduced to 40- or 20-mg after 6 and 12 months on cabozantinib treatment. In general, the simulated data set for 60-mg starting dose showed a similar pattern for dose modification as the observed 60-mg dose data. Slight differences between the observed and simulated data sets may reflect the lack of a drop-out (based on progressive disease or death) in the modeled analysis. Relative to a 60-mg simulated starting dose, a lower percentage of subjects were predicted to have dose-reduced at a 40-mg simulated starting dose at both the 6-month treatment time-point (24 vs 45%) and 12-month treatment time-point (37 vs 64%). At 6 months, approximately 50% of subjects in the 60-mg starting dose group (observed and simulated) are still on the 60-mg dose.

**Fig. 5:**
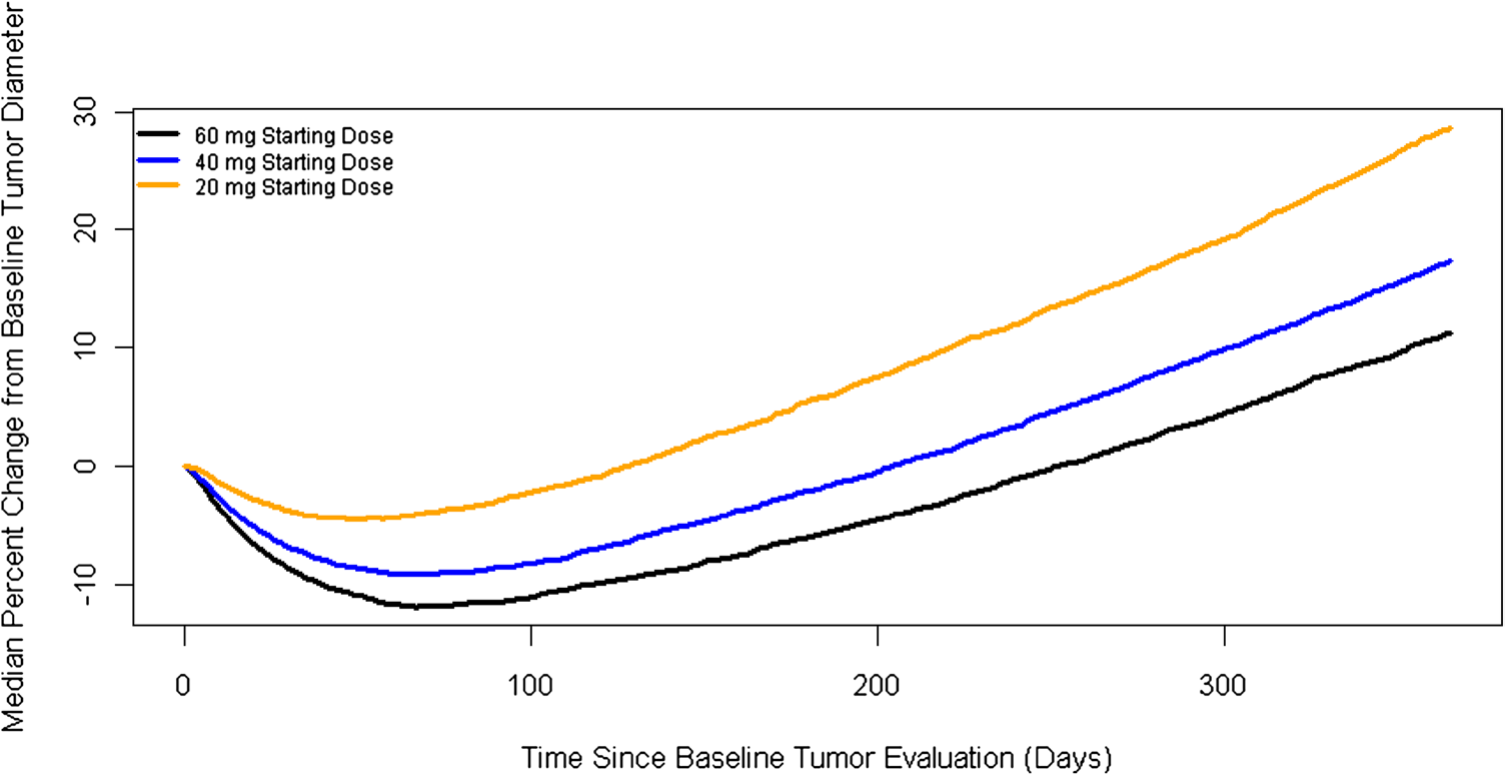
Comparison of predicted median percent change from baseline tumor diameter for 20-, 40-, and 60-mg cabozantinib simulated starting doses

Next, the tumor growth model was used to simulate the time course of tumor diameters for each of the 1000 patients in the 60-, 40-, and 20-mg starting dose treatment groups. Patients in the 20- and 40-mg starting dose treatments groups were predicted to have a smaller reduction (median change from baseline = − 4.45 and − 9.1%, respectively) in tumor size relative to the 60-mg (–11.9%) starting dose treatment group (Fig. 5).

To further assess the clinical relevance of the difference in tumor reduction between starting doses, the response to treatment was computed at baseline and every 8 weeks for 1 year using the longitudinal sum of tumor diameter predictions. The responses [complete response (CR), partial response (PR), stable disease, and progressive disease (PD)] were calculated using the criteria outlined in the METEOR study. In the simulated 60-mg starting dose group, a higher percentage of patients were predicted to achieve objective response (CR + PR) relative to the 40- and 20-mg starting dose groups (19.1, 15.6, and 8.7%, respectively), whereas a lower percentage of patients were predicted to have PD (7.5, 8.1, and 10.2%, respectively; Table 1).

**Table 1:** Percentage of simulated subjects (*N* = 1000) achieving each best overall response category

Best overall response	20-mg starting dose (%)	40-mg starting dose (%)	60-mg starting dose (%)
Complete response	0.10	0.00	0.00
Partial response	8.60	15.6	19.1
Stable disease	81.1	76.3	73.4
Progressive disease	10.2	8.10	7.50

## Discussion

In the phase III METEOR study, cabozantinib demonstrated significantly improved PFS [HR 0.51 (95% CI 0.41–0.62); median 7.4 vs 3.9 months; *p* < 0.0001], ORR [17% (13–22) vs 3% (2–6); *p* < 0.0001], and OS [HR 0.66 (0.53–0.83); median 21.4 vs 16.5 months; *p* = 0.0003] versus everolimus in patients with advanced RCC who had received prior VEGFR-TKI therapy [11]. A high percentage (~ 60%) of RCC patients treated with cabozantinib in METEOR had at least one dose reduction from the 60-mg FBE dose (to 40- or 20-mg FBE) in response to treatment-emergent AEs. ER models were thus developed to describe the relationship between cabozantinib exposures at doses evaluated in METEOR and measures of efficacy and safety (AEs and the need for dose modifications) using data in RCC patients enrolled in the pivotal phase III study.

A statistically significant relationship was identified between the rate of PFS (progressive disease or death) and the time-varying average cabozantinib concentration. Increases in cabozantinib concentration were predicted to decrease the rate of PFS in a nonlinear manner, with an EC_50_ value (100 ng/mL) by the best nonlinear (*E*_max_) model markedly lower than the predicted steady-state average cabozantinib concentrations of the modeled 20-, 40-, and 60-mg dose levels (375, 750 and 1125 ng/mL, respectively). The nonlinear relationship was reflected in the marginally increased HRs (1.10 and 1.39) for risk of progressive disease or death for simulated concentrations at starting doses of 40- and 20-mg, respectively, vs 60-mg.

Covariates that were associated with an increase in rate of disease progression were baseline ECOG score of ≥ 1, baseline tumor diameters above the median, presence of liver metastasis, MET IHC status designated as high, and having a < 3-month time elapsed before disease progression with prior TKI therapy. At clinically relevant cabozantinib concentrations, the effects of the covariates decreased, such that the HRs generally reflected primarily drug effect on PFS. However, subjects that had progressive disease prior to 3 months on the previous TKI therapy showed a higher HR compared to subjects that had progressive disease after 3 months on the previous TKI therapy for the same cabozantinib plasma concentrations and appeared to be minimally affected by increasing cabozantinib plasma concentration.

The nonlinear mixed-effect tumor growth model developed using target lesion tumor diameter measurements from RCC patients administered cabozantinib in METEOR yielded an estimated EC_50_ value (251 ng/mL) lower than the predicted steady-state average cabozantinib concentrations for the starting dose levels of 60-, 40-, and 20-mg (1125, 750, and 375 ng/mL, respectively). The corresponding predicted median percent change of tumor size from baseline (− 11.9, − 9.1, and − 4.5% respectively) and predicted ORR (19.1, 15.6, and 8.7%, respectively) indicates the 60-mg cabozantinib starting dose which provides relatively greater anti-tumor activity compared to those predicted for lower simulated starting doses of 40- and 20-mg. These findings in RCC patients are consistent with those of the nonlinear mixed-effect models developed previously to describe the relationship between cabozantinib exposure and target lesion tumor size in the phase III EXAM study of patients with progressive metastatic MTC: the estimated EC_50_ values (range 58–79 ng/mL) were lower than the steady-state cabozantinib concentration at the 140-mg dose level (1640 ng/mL), and no marked decrease in target lesion regrowth was predicted as a consequence of the two protocol-defined dose reductions to 100- and 60-mg [20, 21].

An increase in cabozantinib concentration was associated with an increased risk of AEs fatigue/asthenia (grade ≥ 3), PPES (grade ≥ 1), hypertension (systolic BP > 160 mmHg or diastolic BP > 100 mmHg), and diarrhea (grade ≥ 3), with predicted HRs for these AEs approximately 1.4- and twofold higher at the predicted steady-state average cabozantinib concentration for a 60-mg dose relative to a 40- or 20-mg dose, respectively. Cabozantinib showed moderately high inter-individual variability for CL/*F* in subjects with various tumor types including RCC based on popPK modeling (percent coefficient of variation = approximately 46% [22]); thus, the 60-mg starting dose would provide RCC subjects with higher cabozantinib CL/*F* (and lower exposures relative to subjects with lower CL/*F*) the opportunity to achieve therapeutic concentrations. For RCC subjects with lower cabozantinib CL/*F*, dose modification to 40- or 20-mg provides the opportunity to achieve a tolerated plasma exposure that also yields acceptable clinical activity.

Treatment-emergent AEs frequently lead to dose modifications in RCC patients enrolled in METEOR. Patients were permitted to modify or interrupt treatment at non-uniform times over the course of the METEOR study, so a repeated time-to-event model was developed that incorporated all dose changes (dose holds, reductions and increases) over time and was used to calculate time-varying cabozantinib exposure based upon the dose changes.

While simulations showed that the 20-, 40-, and 60-mg cabozantinib starting dosages were all predicted to reduce tumor growth, the 60-mg dose resulted in the greatest reduction in tumor growth, best ORR, and lowest rate of PD. In the review of the New Drug Application for cabozantinib for the treatment of patients with RCC [23], the Food and Drug Administration (FDA) addressed the issue regarding the appropriateness of dose selection given the high percentage of dose reductions in METEOR. Based on their review, FDA concluded that: (1) most adverse reactions were successfully managed with dose interruptions and supportive measures; (2) ER modeling indicated that lower starting doses could possibly compromise activity of the drug with decreased response rates; and (3) the dose selection of 60-mg daily was adequate based on ER analyses and a safety profile that is acceptable for the patient population. The cabometyx label specifies dose reduction instructions for the RCC population [12].

## Conclusions

In ER models, a 60-mg simulated starting dose resulted in improved PFS, reduced tumor growth, and increased ORR compared to a 40- or 20-mg simulated starting dose. However, higher cabozantinib exposures resulting from lower cabozantinib CL/*F* are predicted to increase the rate of dose modification, while reducing cabozantinib exposure with dose reduction is projected to decrease the risk of individual clinically relevant AEs in RCC patients. Overall, the ER analysis predicts that cabozantinib would be effective at the 60-mg starting dosage evaluated in METEOR as well as daily dosages of 40- and 20-mg that resulted from dose reduction.

## Electronic supplementary material

Below is the link to the electronic supplementary material.

Supplementary material 1 (DOCX 14 KB)

Supplementary material 2 (DOCX 16 KB)

Supplementary material 3 (DOCX 16 KB)

Supplementary material 4 (DOCX 16 KB)
