# Supplementary material for: Population exposure–response analysis of cabozantinib efficacy and safety endpoints in patients with renal cell carcinoma
Source: Cancer Chemother Pharmacol. 2018 Apr 17;81(6):1061–70. doi: 10.1007/s00280-018-3579-7 (PMC5973957; doi:10.1007/s00280-018-3579-7)
Supplement: Supplementary file 1 — Supplementary material 1 (DOCX 14 KB) [file 280_2018_3579_MOESM1_ESM.docx]

**Supplemental** **Table 1. Summary of Number of Events and Number of Subjects at Risk for Time-to-Event Endpoints**

| **End Point** | **Number of Events for Subjects^a^ with at least** | | **Total Number of Subjects at Risk with at least** | |
| --- | --- | --- | --- | --- |
|  | **One dose of cabozaninib** | **One measureable cabozantinib concentration** | **One dose of cabozaninib** | **One measureable cabozantinib concentration** |
| Progression Free Survival | 179 | 172 | 323 | 315 |
| Fatigue/Asthenia^b^ | 43 | 42 | 329 | 318 |
| Palmar-Plantar Erythrodysesthesia^c^ | 139 | 137 | 329 | 318 |
| Nausea/Vomiting^b^ | 16 | 16 | 329 | 318 |
| Diarrhea^b^ | 38 | 38 | 329 | 318 |
| Hypertension^d^ | 105 | 103 | 328 | 318 |
| Stomatitis^b^ | 10 | 10 | 329 | 318 |

^a^One event counted per endpoint per subject.

^b^Grade≥3

^c^Grade≥1

^d^Systolic blood pressure > 160 mmHg or diastolic blood pressure > 100 mmHg

.
