# Supplementary material for: Population exposure–response analysis of cabozantinib efficacy and safety endpoints in patients with renal cell carcinoma
Source: Cancer Chemother Pharmacol. 2018 Apr 17;81(6):1061–70. doi: 10.1007/s00280-018-3579-7 (PMC5973957; doi:10.1007/s00280-018-3579-7)
Supplement: Supplementary file 2 — Supplementary material 2 (DOCX 16 KB) [file 280_2018_3579_MOESM2_ESM.docx]

**Supplemental Table 2.** **Summary of Covariate Data**

| **Covariate** | **Measurement** | **N** | **%** |
| --- | --- | --- | --- |
| **Baseline ECOG Score** | Missing | 9 | 2.74 |
|  | 0 | 164 | 49.85 |
|  | 1 | 150 | 45.59 |
|  | 2 | 6 | 1.82 |
| **Number of MSKCC Risk Factors** | 0 (favorable prognosis) | 150 | 45.59 |
|  | 1 (intermediate prognosis) | 137 | 41.64 |
|  | 2 or 3 (poor prognosis) | 42 | 12.77 |
| **Baseline Sum of Diameter^a^** | <Median | 165 | 50.15 |
|  | ≥Median | 164 | 49.85 |
| **Visceral and Bone Metastases** | No | 269 | 81.76 |
|  | Yes | 60 | 18.24 |
| **Lung Metastases** | No | 125 | 37.99 |
|  | Yes | 204 | 62.01 |
| **Liver Metastases** | No | 241 | 73.25 |
|  | Yes | 88 | 26.75 |
| **Bone Metastases** | No | 252 | 76.60 |
|  | Yes | 77 | 23.40 |
| **Prior No. of VEGF-target TKI** | 1 | 232 | 70.52 |
|  | 2 or more | 97 | 29.48 |
| **Number of Organs Involved^a^** | Missing | 2 | 0.61 |
|  | 1 | 59 | 17.93 |
|  | 2 | 101 | 30.70 |
|  | ≥3 | 167 | 50.76 |
| **Time to Progressive Disease for Most Recent Prior TKI** | Missing | 3 | 0.91 |
|  | <3 months | 44 | 13.37 |
|  | ≥3 months | 282 | 85.71 |
| **Received only Prior Sunitinib** | No | 195 | 59.27 |
|  | Yes | 134 | 40.73 |
| **Received only Prior Pazopanib** | No | 241 | 73.25 |
|  | Yes | 88 | 26.75 |
| **Prior Agents targeting PD-1** | No | 311 | 94.53 |
|  | Yes | 18 | 5.47 |
| **Prior Nephrectomy** | No | 47 | 14.29 |
|  | Yes | 282 | 85.71 |
| **Tumor MET IHC Status** | High | 48 | 14.59 |
|  | Low | 138 | 41.95 |
|  | Unknown | 143 | 43.47 |

*ECOG* Eastern Cooperative Oncology Group, *IHC* immunohistochemistry, *MET* hepatocyte growth factor receptor protein, *MSKCC* Memorial Sloan Kettering Cancer Center, *PD-1* programmed cell death immune receptor, *TKI* tyrosine kinase inhibitor, *VEGFR* vascular endothelial growth factor receptor,

^a^As determined per Independent Radiology Committee
