# Supplementary material for: Population exposure–response analysis of cabozantinib efficacy and safety endpoints in patients with renal cell carcinoma
Source: Cancer Chemother Pharmacol. 2018 Apr 17;81(6):1061–70. doi: 10.1007/s00280-018-3579-7 (PMC5973957; doi:10.1007/s00280-018-3579-7)
Supplement: Supplementary file 3 — Supplementary material 3 (DOCX 16 KB) [file 280_2018_3579_MOESM3_ESM.docx]

**Supplemental Table 3.** **Parameter Estimates for the Exposure-Response Model for Sum of Tumor Diameter, Dose Reduction/Interruption and All Dose Modification**

| **Model** | **Transformed Parameter Description** | **Transformed Estimate^a^** | **Transformed 90% CI** |
| --- | --- | --- | --- |
| Longitudinal Tumor Growth | Baseline Tumor Size (mm) | 63.1 | 58.9, 67.5 |
|  | *k_grow_* (1/day) | 0.00155 | 0.00133, 0.0018 |
|  | *k_dmax_* (1/day) | 0.00125 | 0.000984, 0.00158 |
|  | *k_dmax_tot_* (1/day) | 0.00835 | 0.00689, 0.0101 |
|  | EC_50_ (ng/mL) | 251 | 169, 375 |
|  | *k_tol_* (1/day) | 0.0271 | 0.0238, 0.0308 |
|  | Residual Variability (SD) (mm) | 5.75 | 5.52, 6 |
|  | IIV Base (ω) | 0.522 | 0.45, 0.594 |
|  | IIV k_grow_ (ω) | 0.313 | 0.218, 0.408 |
|  | IIV k_dmax_ (ω) | 0.353 | 0.224, 0.482 |
|  | IIV k_dmax_tol_ (ω) | 0.641 | 0.469, 0.814 |
|  | IIV EC_50_ (ω) | 0.02^b^ | 0.02. 0.02 |
|  | IIV k_tol_ (ω) | 0.02^b^ | 0.02. 0.02 |
|  |  |  |  |
| All Dose Modification Repeated Time to Event (DMAK model) | *θ_base_* | –5.4 | –5.6, –5.2 |
|  | *θ_drug_* | 0.000807 | 0.000644, 0.000969 |
|  | *θ_base-hold_* | –2.7 | –2.82, –2.57 |
|  | IIV baseline | 0.655 | 0.507, 0.803 |

CI confidence interval, EC_50_ Concentration achieving one-half of maximal effect, *IIV* inter-individual variability, *k_dmax_*  maximum non-attenuating tumor decay rate, *k_dmax_tot_*  maximum attenuating tumor decay rate, *k_grow_* tumor growth rate, *k_tol_* attenuation rate constant, *SD* standard deviation, *θ* baseline log hazard, *θ_drug_* change in log hazard per unit cabozantinib concentration, *θ_base-hold_* baseline log hazard for dose hold
^a^ Units reflect values for transformed parameters; ^b^ fixed value
