# Supplementary material for: Population exposure–response analysis of cabozantinib efficacy and safety endpoints in patients with renal cell carcinoma
Source: Cancer Chemother Pharmacol. 2018 Apr 17;81(6):1061–70. doi: 10.1007/s00280-018-3579-7 (PMC5973957; doi:10.1007/s00280-018-3579-7)
Supplement: Supplementary file 4 — Supplementary material 4 (DOCX 16 KB) [file 280_2018_3579_MOESM4_ESM.docx]

**Supplemental Table 4. Parameter Estimates for the Final Cox Proportionality Hazard Models in Patients with Renal Cell Carcinoma Model**

|  | **Parameter** | **Estimate** | **Standard Error** | **Likelihood Ratio (χ^2^)** | **p-value** |  |  |  |  |  |
| --- | --- | --- | --- | --- | --- | --- | --- | --- | --- | --- |
| Progression Free Survival | β_TR-CAVG3W_ | –1.25581 | 0.63139 | 3.9559 | 0.0467 |  |  |  |  |  |
|  | β_ECOG Score (≥1)_ | 0.85058 | 0.50374 | 2.8512 | 0.0913 |  |  |  |  |  |
|  | β_TR-CAV3W*ECOG Score (≥1)_ | –0.31399 | 0.61166 | 0.2635 | 0.6077 |  |  |  |  |  |
|  | β_SOD(>Median)_ | 0.84555 | 0.49149 | 2.9597 | 0.0854 |  |  |  |  |  |
|  | β_TR-CAV3W*_β_SOD (>median)_ | –1.25269 | 0.59989 | 4.3606 | 0.0368 |  |  |  |  |  |
|  | β_LIVER METS (yes)_ | 1.32190 | 0.50495 | 6.8534 | 0.0088 |  |  |  |  |  |
|  | β_TR-CAV3W*_β_LIVER METS (yes)_ | –1.26391 | 0.62994 | 4.0256 | 0.0448 |  |  |  |  |  |
|  | β_ICH MET (high)_ | 1.18288 | 0.59877 | 3.9027 | 0.0482 |  |  |  |  |  |
|  | β_TR-CAVG3W*_β_ICH MET (high)_ | –1.63127 | 0.76851 | 4.5056 | 0.0338 |  |  |  |  |  |
|  | β_TR-CAVG3W*_β_Prior TKI PD(<3mon)_ | 0.81809 | 0.24775 | 10.9041 | 0.0010 |  |  |  |  |  |
| Fatigue/Asthenia | β_CAVG2W_ | 0.0009319 | 0.000338 | 6.9453 | 0.0084 |  |  |  |  |  |
| Palmar-Plantar Erythrodysesthesia | β_CAVG2W_ | 0.00106 | 0.0002208 | 21.8481 | <0.0001 |  |  |  |  |  |
| Nausea/Vomiting | β_CAVG2W_ | 0.00104 | 0.0005360 | 3.4415 | 0.0636 |  |  |  |  |  |
| Diarrhea | β_CAVG2W_ | 0.0007653 | 0.0003351 | 4.847 | 0.0277 |  |  |  |  |  |
| Stomatitis | β_CAVG0T_ | 0.00169 | 0.0008533 | 3.5477 | 0.0596 |  |  |  |  |  |
| Hypertension | β_CAVGID_ | 0.0008187 | 0.0002234 | 12.9142 | 0.0003 |  |  |  |  |  |

β_TR-CAVG3W_*,* the maximum log hazard ratio for average concentration of cabozantinib calculated over the three weeks prior to time of response measure for PFS, *CAVG2W* average concentration of cabozantinib calculated over the two weeks prior to time of response measure,*CAVG3W* average concentration of cabozantinib calculated over the three weeks prior to time of response measure, *CAVG0T* the average cabozantinib concentration from time zero to the event, *CAVGID* cabozantinib concentration calculated over the 24 hours prior to time equal t, *for AE endpoints,* β is the change in the log hazard ratio per unit change in each exposure metric; *ECOG,* Eastern Cooperative Oncology Group*, IHC* immunohistochemistry, *LIVER METS* Liver metastasis, *MET* hepatocyte growth factor, *SOD* sum of diameter of tumor, *TKI PD,* Progressive disease on prior tyrosine kinase inhibitor
